# Arthroscopy-assisted partial trapeziectomy combined with ligament reconstruction for thumb carpometacarpal joint osteoarthritis: A different technique

**DOI:** 10.3389/fsurg.2022.945013

**Published:** 2022-09-12

**Authors:** Wan-Ling Zheng, Yang-Chun Wu, Yun-Dong Shen, Hua-Wei Yin, Wen-Dong Xu

**Affiliations:** ^1^ Department of Hand Surgery, Huashan Hospital, Fudan University, Shanghai, China; ^2^ Department of Hand and Upper Extremity Surgery, Jing’an District Central Hospital, Shanghai, China; ^3^ National Clinical Research Center for Aging and Medicine, Huashan Hospital, Fudan University, Shanghai, China; ^4^ Priority Among Priorities of Shanghai Municipal Clinical Medicine Center, Shanghai, China; ^5^ Institute of Engineering and Application Technology, Fudan University, Shanghai, China

**Keywords:** thumb, carpometacarpal joint (CMC), osteoarthritis, arthroscopy, ligament reconstruction and tendon interposition, trapeziectomy

## Abstract

**Background:**

There is no consensus on the best surgery option for thumb carpometacarpal osteoarthritis (CMC OA). The traditional method has the risk of large trauma, obvious metacarpal subsidence, and decreased stability. The aim of this study is to introduce a different technique to restore the function and stability of the first carpal metacarpal joint with minimal trauma, rapid pain relief, reduced complications, and the clinical outcomes in the long-term follow-up was evaluated and statistically analyzed.

**Methods:**

This was a retrospective study of 10 patients with a mean age of 51.8 years. The surgery consisted of removing partial trapezium through arthroscopy, reconstructing the stability with flexor carpi radialis suspension and tendon interposition. The subjective assessment included visual analog scale (VAS) of pain, quick disabilities of the arm, shoulder, and hand (Quick-DASH) score, and patient satisfaction. The range of motion, grip strength, pinch strength, and radiographic assessment, which can reflect stability of the thumb, were objectively evaluated and statistically analyzed.

**Results:**

Ten patients were monitored at a mean follow-up of 6.8 years. The mean grip strength improved significantly from 16.64 to 22.57 kg after surgery. Pinch strength improved significantly from 3.72 to 5.71 kg on average. The Kapandji score improved signiﬁcantly from 5.7 to 8.6 on average. 80% (8/10) of the patients were satisfied with this surgery. On objective indicators, the VAS score decreased signiﬁcantly from 6.4 to 1.3 on average. The mean Quick-DASH score improved signiﬁcantly from 6.1 to 28.9. Postoperative x-ray showed slight subsidence and dislocation of the first metacarpal in two patients and did not affect the function by measurement.

**Conclusion:**

Arthroscopy-assisted partial trapezium resection combined with ligament reconstruction could be a workable and promising surgical technique in patients with thumb CMC OA. It can offer the advantages of minimizing surgical injury by preserving the first carpal metacarpal joint capsule to protect its stability, with a rapid pain relief, function improvement, and satisfactory results in patients’ clinical measurements.

## Introduction

One out of three people aged 55 years and older have radiographic signs of carpometacarpal osteoarthritis (CMC OA) (1–3). Patients with mild symptoms in stage I and II can wear braces to increase stability and reduce mechanical stress. Patients of stage II to stage IV, in whom symptoms remain despite conservative treatment, should be treated by operation. There are numerous options in surgical treatment, such as trapeziectomy, ligament reconstruction, and tendon interposition or suspension arthroplasty (4–6). The traditional open trapeziectomy will destroy the joint capsule and affect the stability of the joint. Second, the classical flexor carpi radialis tendon reconstruction bypasses itself and is sutured and fixed in the first metacarpal bone, which will produce a downward pulling force during movement and may aggravate the sinking of the first metacarpal bone. In recent years, many studies have focused on new methods of tendon and ligament reconstruction, as well as minimally invasive surgery under wrist arthroscopy. However, there is still no consensus on the optimal treatment.

Compared with previous studies, this article introduced a different technique of arthroscopy-assisted partial trapezium resection, combined with ligament reconstruction and tendon interposition at the same time. This method not only reduces the damage to the capsular ligament and reduces the trauma but also partially maintains the height and stability of the metacarpal bone, which can be applied to more complex cases. Specifically, arthroscopy-assisted partial trapezium resection is less invasive compared with the parallel cross section of traditional open surgery. Moreover, the ball-and-socket joint was innovatively proposed in the surgery of the first carpal and metacarpal joint in this study. By modifying the saddle shape of the first carpal metacarpal joint into ball-and-socket joint, it may be more consistent with joint physiology and more stably adapt to the multidirectional activity requirements of the first carpal metacarpal joint. Finally, the classical approach of wrapping around the flexor carpi radialis has a downward force, which may aggravate the sinking of the first metacarpal. In our surgery, the approach of suturing around the first metacarpal itself creates an upward lifting force, theoretically reducing the sinking of the metacarpal.

The aim of this study was to introduce this different surgery, and the clinical outcomes in the long-term follow-up were evaluated and statistically analyzed.

## Methods

### Subjects

**Figure 1:**
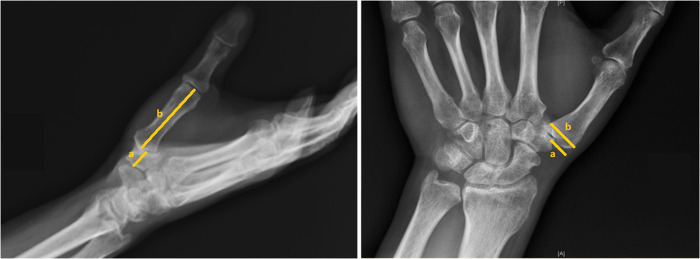
Radiograph of CMC OA with loss of joint space and radial dislocation. (Left) (a): axis of the trapezium and (b): axis of the first metacarpal; (right) (a): uncovered length of trapezium and (b): total length of trapezium.

From January 2012 to December 2021, 13 patients with thumb CMC arthritis were empirically selected and treated with this method, out of which 3 patients were lost to follow-up, leaving 10 patients (5 males and 5 females) for analysis. The indication criteria were patients with thumb CMC OA in stage II and stage III, combined with metacarpal dislocation, which required tendon and ligament reconstruction. One patient in stage IV strongly requested to attempt this surgery to delay the progression of arthritis due to the risk of metacarpal subsidence and instability after total trapeziectomy. All patients had clinical symptoms after at least 3 months of conservative treatment, confirmed by x-ray examination (Figure 1). According to Eaton staging (7), there were five patients in stage II, four patients in stage III, and one patient in stage IV.

### Surgical procedure

Under general anesthesia, the patient was supine on an operating table with an arm board. A tourniquet (pressure: 34 kPa, limit time: 60 min) and wrist joint distraction of 15 lb were applied.

### Stage I: arthroscopy-assisted partial trapezium resection

**Figure 2:**
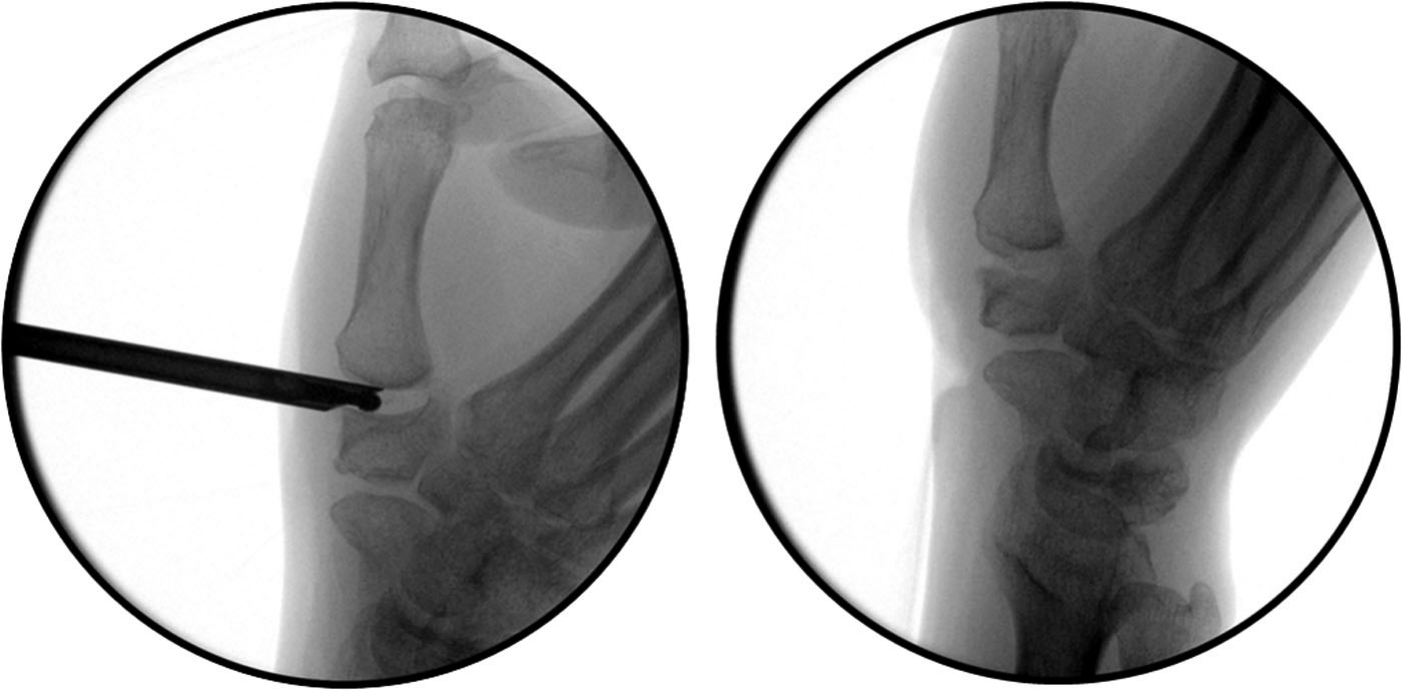
Arthroscopy-assisted partial trapezium resection. (Left): Partial trapeziectomy is performed with the 1.9 mm burr. (Right): The articular surface of the part of the trapezium is removed until it shows hemispherical fossa.

In the ﬁrst step, two 4 mm transverse portals are made along the dorsoradial border of the hand with the thumb CMC joint as the center. The viewing camera is placed on the dorsoradial 1R portal to explore the articular surface; the shaver and radiofrequency are introduced in the dorsoulnar 1U portal to remove the hyperplastic synovium in the first CMC joint. The scaphoid trapezium trapezoid joint was explored and treated with the same method if needed. Then, arthroscopic partial resection of the trapezium is performed. Partial trapeziectomy is performed with the 1.9 mm burr, and part of the articular surface is removed until it shows hemispherical fossa of 3 mm depth (Figure 2). We polish the saddle joint and create a space for a tendon ball (Supplementary Video S1). The spur is resected to improve the range of motion (ROM) and symptoms as well as increase the stability of the basal joint.

### Stage II: ligament reconstruction

**Figure 3:**
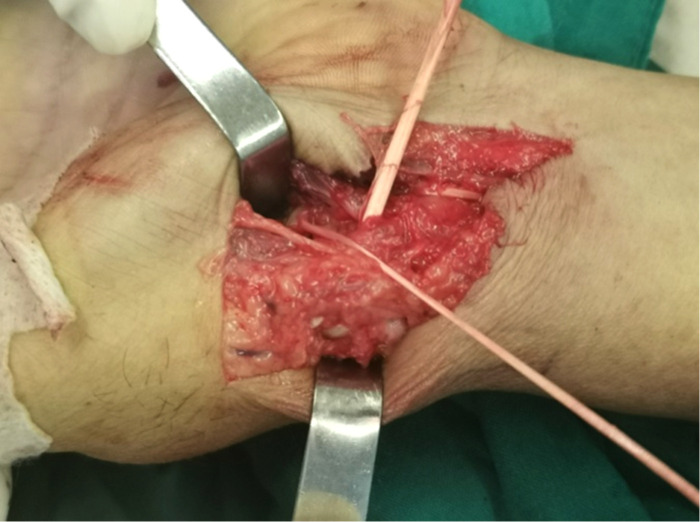
The radial half of the tendon is transected and dissociated distally to the level of the CMC joints.

A transverse incision is made at approximately 5 cm level of the transverse wrist lines to expose the flexor carpi radialis. The radial half of the tendon is transected and dissociated distally to the level of the CMC joints (Figure 3). A 3.0 mm k-wire is drilled gradually to expand the bone hole. Then, the strip of the ﬂexor carpi radialis is passed from the palmar side through the transosseous tunnel in the first metacarpal to the dorsal side, and the first metacarpal bone is fixed to the second metacarpal bone with a 1.5 mm k-wire. After wrapping around the base of first metacarpal, the spare flexor tendon is sutured with itself on the volar side. The remaining tendons are sutured to form a tendon ball.

**Figure 4:**
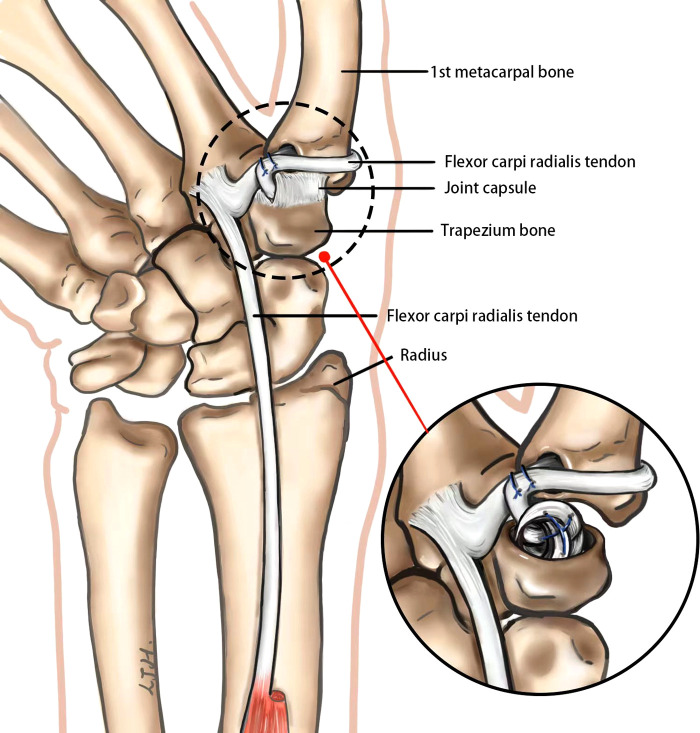
Ligament reconstruction. Surrounding the base of first metacarpal, one half of the tendon passes from the volar side to the dorsal side through the transosseous tunnel in first metacarpal and sutures with itself on the volar side.

A grasper was inserted into the CMC joint from the 1R portal, which came out from the volar portal, to take the tendon ball from palmer side into the CMC joint space. Suture was used to fix the tendon ball to the dorsal side of joint capsule to prevent displacement. Large incision of the thumb CMC capsule is avoided during the whole procedure (Figure 4).

### Postoperative management

**Figure 5:**
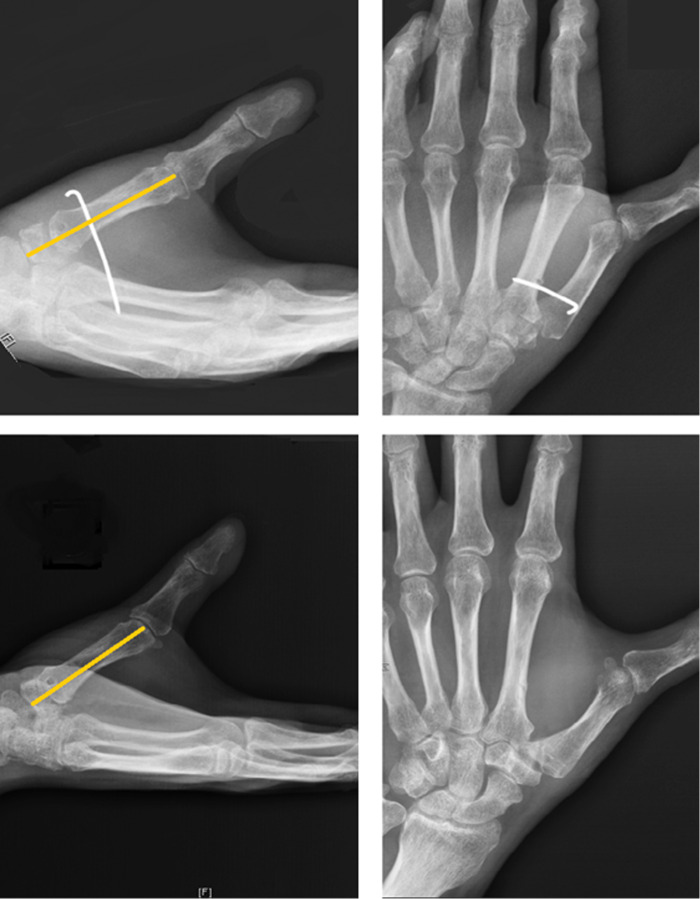
Fluoroscopy shows a good position with slight subsidence of the first metacarpal bone. (Above): Two months after surgery. (Below): Six months after surgery.

After the surgery, a short-arm cast is fixed for 4 weeks, followed by a removable splint for another 4 weeks. The Kirschner wire was removed after 2 months (Figure 5). Then, patients were allowed unrestricted use of the wrist.

### Postoperative evaluation

Patients were followed up at 2 months, 6 months, and every year after surgery. Clinical data were collected by an independent examiner. As compared with the preoperative condition and contralateral side, the clinical evaluation studied the ROM of the first column according to the Kapandji score (8). Grip strength was measured in kg using a Jamar (9) dynamometer, and pinch strength were measured in kg using the Preston (10) pinchmeter. Radiographs were taken in anterior and lateral planes before and after surgery. They were screened for dislocation, subsidence, and adjacent joint OA and can be used to assess joint stability (11).

At the last follow-up visit before summary, the pain intensity was evaluated on a visual analog scale (VAS) (12) from 0 (no pain) to 10 (worst imaginable pain). The ability to carry out daily life activities was evaluated based on the quick disabilities of the arm, shoulder, and hand (Quick-DASH) (13) questionnaire. Subjective evaluation of satisfaction was done with an analytical questionnaire ranging from 0 (dissatisfaction) to 10 (great satisfaction). In addition, the patient’s impression of thumb stability and ROM were evaluated by subjective as compared with the preoperative condition.

### Statistical analysis

Data are presented as the mean ± standard deviation and 95% confidence intervals. The group means were compared using analysis of Student’s *t* test. Statistical analysis was performed using SPSS 20 and *p* < 0.05 was recognized as significant.

## Results

All patients underwent surgery in the same center between January 2012 and December 2021. The average age at admission was 51.8 years (range: 33–62). Mean follow-up was 6.8 years (range: 10–128 months). Preoperative characteristics are summarized in Table 1.

**Table 1:** Demographics of patients at baseline.

Number	Sex	Age	Injured hand	Eaton stage	Duration time (months)	Dominant hand	Causes
1	Male	53	Right	II	6	Right	Injury
2	Male	53	Left	II	4	Right	Degenerative
3	Female	59	Left	II	3	Right	Rheumatoid
4	Female	55	Left	III	6	Right	Degenerative
5	Female	62	Right	III	6	Left	Unknown cause
6	Female	57	Right	II	48	Right	Injury
7	Male	33	Right	III	24	Right	Degenerative
8	Male	39	Right	II	9	Right	Injury
9	Male	48	Left	IV	12	Right	Injury
10	Female	59	Left	III	12	Right	Degenerative

First, the 1-year follow-up data were analyzed in Table 2. Compared to the preoperative condition, the grip strength improved significantly to an average of 22.57 kg (*p* = 0.003). There was no significant difference relative to the grip strength in the healthy hand, which was 24.61 kg on average (*p* = 0.29). The postoperative pinch strength was also better than preoperative condition (3.72 kg preoperative, 5.71 kg postoperative, *p* = 0.04). Range of motion improved in the Kapandji score (preoperative at 5.70, postoperative at 8.60, *p* < 0.001).

**Table 2:** Comparison of objective outcomes in the preoperative period and 1-year follow-up.

Hand measurements	Side	Mean	Difference
Grip strength, kg	Injury hand (pre)	16.64 ± 1.22 95% CI (13.88–19.40)	*P*_(pre/post) _= 0.003 95% CI (0.10–3.88)
	Injury hand (post)	22.57 ± 1.23 95% CI (19.79–25.35)	
	Healthy hand	24.61 ± 1.42 95% CI (21.41–27.81)	*P*_(I/H) _= 0.29
Pinch strength, kg	Injury hand (pre)	3.72 ± 0.54 95% CI (2.12–4.88)	*P*_(pre/post) _= 0.04 95% CI (2.29–9.57)
	Injury hand (post)	5.71 ± 0.71 95% CI (4.10–7.32)	
	Healthy hand	5.98 ± 0.68 95% CI (4.45–7.51)	*P*_(I/H) _= 0.79
ROM, Kapandji score	Injury hand (pre)	5.70 ± 0.56 95% CI (4.44–6.96)	*P*_(pre/post) _< 0.001 95% CI (1.53–4.27)
	Injury hand (post)	8.60 ± 0.34 95% CI (7.83–9.37)	
	Healthy hand	9.1 ± 0.23 95% CI (8.57–9.628)	*P*_(I/H) _= 0.24

*P*_(pre/post)_ means the difference between injury hand preoperative and postoperative. *P*_(I/H)_ means the difference between injury hand and healthy hand.

Postoperative x-ray showed good joint position with neat axis alignment. Except for one patient who needed joint replacement due to severe polyarthritis of unknown cause, there was no complication like radial nerve injury, tendon rupture, tendinitis, or adjacent joint OA. At 1-year follow-up, two patients had slight metacarpal subsidence and dislocation, which did not affect the function according to the measurement.

**Figure 6:**
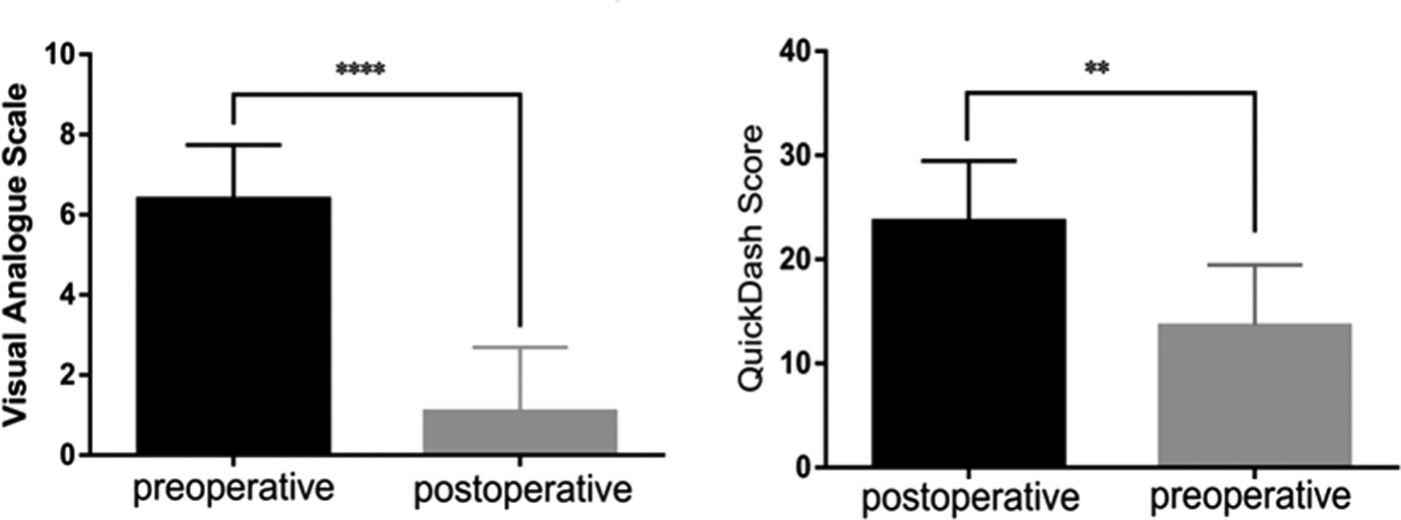
(Left) VAS values for pain. (Right) Quick-DASH score outcomes. **p = 0.001 and ****p < 0.0001.

At the ﬁnal assessment, the pain was significantly decreased in all patients, the mean VAS decreased from 6.4 ± 1.3 to 1.1 ± 1.6 (*p* < 0.001), and the dexterity of the injury hand recovered well with a mean Quick-DASH score increased from 6.1 ± 13.2 to 28.9 ± 13.2 (*p* = 0.001). All patients reported a significant reduction in pain, and the Quick-DASH score was significantly increased than the preoperative condition (Figure 6). Meanwhile, thumb stability and ROM were also greatly improved than the preoperative condition. 80% (8/10) of the patients were satisfied with this surgery, and the mean satisfaction rating score was 8.8 ± 1.7. Detailed results are summarized in Table 3.

**Table 3:** Comparison of subjective outcomes in the preoperative period and the last follow-up.

Number	Follow-up time (months)	VAS (pre/post)	Quick-DASH (pre/post)	Thumb stability	ROM	Satisfaction score
1	128	8/2	13/25	Unstable	Improved	8
2	94	7/0	11/26	Stable	Normal	10
3	95	5/0	11/22	Stable	Improved	10
4	88	5/0	11/23	Stable	Normal	10
5	100	8/5	30/38	Stable	Limited	5
6	88	5/1	13/16	Improved	Normal	10
7	57	5/0	11/20	Stable	Normal	7
8	57	7/2	13/23	Stable	Improved	9
9	10	6/1	12/20	Stable	Improved	9
10	107	8/0	12/24	Stable	Normal	10

VAS, visual analog scale; Quick-DASH, quick disabilities of the arm, shoulder, and hand; ROM, range of motion.

## Discussion

Following the interphalangeal joints, the thumb CMC joint is the second most frequent site of hand OA (14–17). There are numerous options in surgical treatment for CMC OA, with evidence for pain relief and function improved (18). However, comparative studies on different methods and techniques have failed to clarify which techniques are best for treating CMC OA (19). In most hand surgery departments, trapeziectomy combined with ligament reconstruction and tendon interposition is still the most commonly performed technique (20). Compared to trapeziectomy alone, partial resection partially maintained the height of the first metacarpal, and the use of ligamentoplasty adds stability and hence decreases articular irritation (21). Moreover, tendon interposition provides a good buffer while maintaining height, which also helps delay the progression of arthritis. In addition, the advantages of arthroscopy are minimal trauma, rapid recovery, clear field exposure, and its convenience to remove the spur between first and second metacarpal base area to improve symptoms. Although the difficulty of the operation is slightly increased, the operation can be simplified according to the patient’s condition, and the application scope is wider, which has the opportunity to become the gold standard of such surgery.

The thumb CMC joint is known as the saddle joint; this shallow biconcave–convex saddle shape provides little bony stability, and thus the joint relies heavily on its flexible capsular ligaments to prevent subluxation and allow a wide arc of motion (22–24). The main advantage of our procedure is that the large incision of the thumb CMC capsule is avoided. Previous method of partial trapeziectomy is to open the capsule and make the partial trapeziectomy with oscillating saw to form a plain surface. In this procedure, a 1.9 mm burr was used to accomplish the partial trapeziectomy through small portal to minimize the injury to capsule. In addition, hemispherical fossa might be more suitable to hold the tendon ball rather than a plain surface. Although it partially changes the physiology of the joint, it has the opportunity to become a new surgical concept for the treatment of joint diseases and has the potential to be further explored. For the tendon ball interposition step, a grasper is used to take the tendon ball into the joint space. Thus, large incision of the thumb CMC capsule is avoided during the whole procedure. These modifications make it possible to preserve the joint capsule to protect joint instability.

The results of our research have shown that this technique can significantly improve patients’ clinical measurements, functional scores, and relieve patients’ pain with a low complication rate. Although two patients had slight metacarpal subsidence and dislocation, it did not affect function by measurement. This finding is consistent with a recent study which concluded that there was no relationship between postoperative first metacarpal subsidence and functional outcomes (25). The surgical precautions are as follows: (1) Blunt dissection to prevent damage to the radial nerves and CMC capsule; (2) The bone holes should be smooth to prevent the tendon from breaking under stress; (3) The spur should be resected to improve ROM and symptoms and also increase the stability of basal joint; (4) The tendon ball is sutured to the dorsal side of the capsule to prevent displacement.

The study has some limitations. First, our study is a retrospective long-term review, which may include the inherent bias and underestimated the complication rate (26). To overcome the lack of a comparison group for there is no gold standard procedure for this disease, we compared data with preoperative and healthy side. Because the recovery effect was well, further research with a larger sample size is needed for verification, and further horizontal comparison of different surgical methods to screen out the gold standard CMC OA treatment is meaningful.

## Conclusions

This long-term follow-up study indicates that arthroscopy-assisted partial trapezium resection combined with ligament reconstruction could be a workable and promising surgical technique in patients with thumb CMC OA. It can offer the advantages of minimizing surgical injury by preserving the first carpal metacarpal joint capsule to protect its stability, with rapid pain relief, function improvement, and satisfactory results in patients’ clinical measurements.

## Data Availability

The raw data supporting the conclusions of this article will be made available by the authors, without undue reservation.
