# Supplementary figures and images for: Arthroscopy-assisted partial trapeziectomy combined with ligament reconstruction for thumb carpometacarpal joint osteoarthritis: A different technique
Source: Front Surg. 2022 Sep 12;9:945013. doi: 10.3389/fsurg.2022.945013 (PMC9632966; doi:10.3389/fsurg.2022.945013)

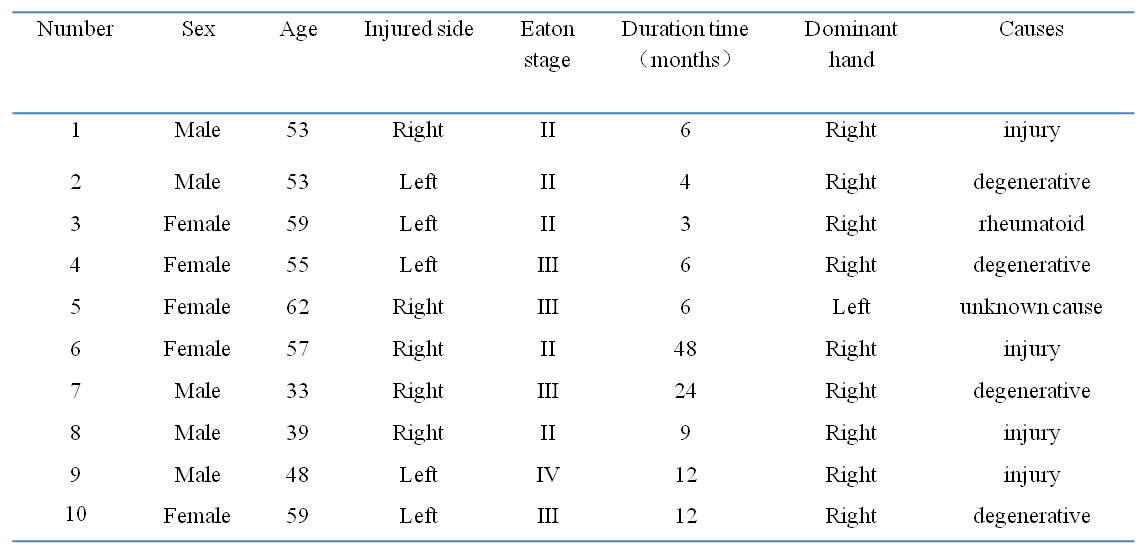

Supplement: Supplementary file 1 [file Image1.tif]

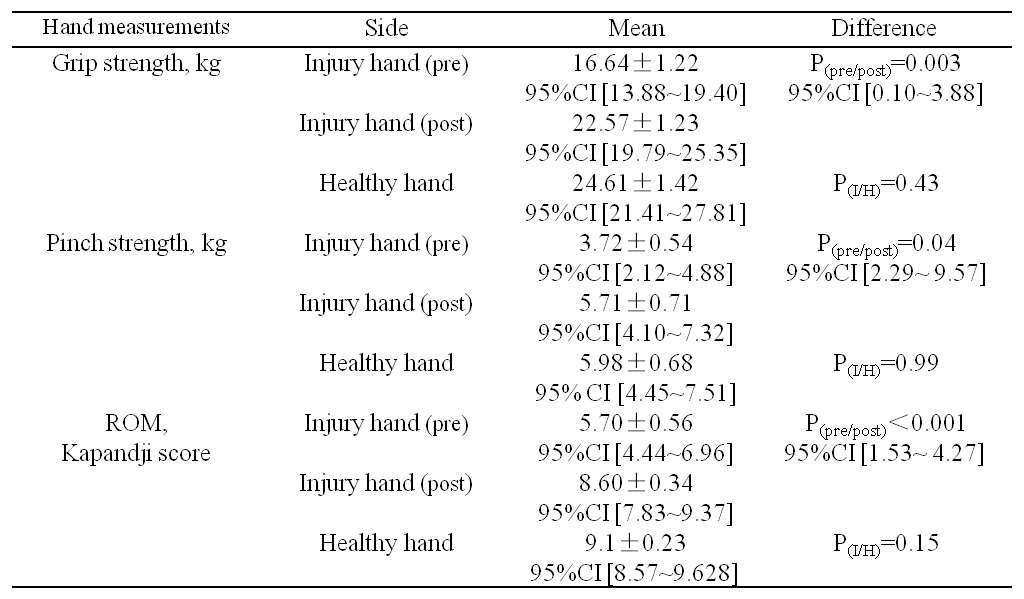

Supplement: Supplementary file 2 [file Image2.tif]

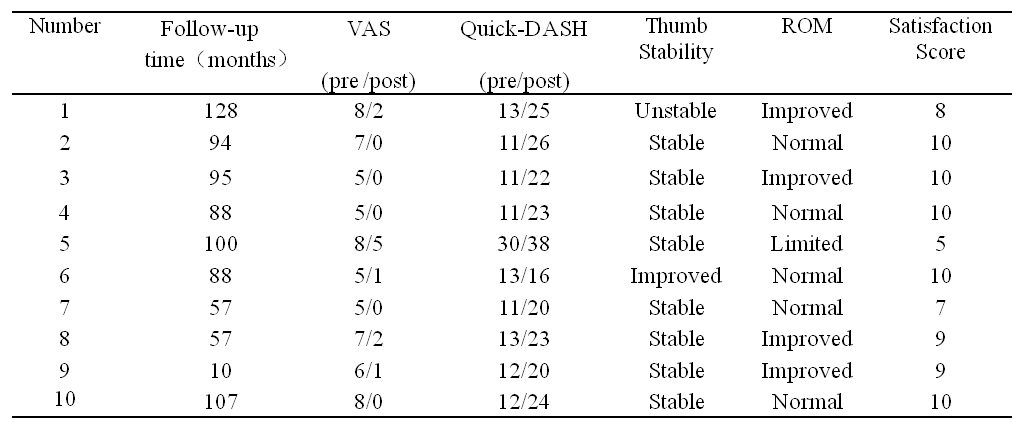

Supplement: Supplementary file 3 [file Image3.tif]
